# Resistance to dual-gene Bt maize in *Spodoptera frugiperda*: selection, inheritance, and cross-resistance to other transgenic events

**DOI:** 10.1038/srep18243

**Published:** 2015-12-17

**Authors:** Oscar F. Santos-Amaya, João V. C. Rodrigues, Thadeu C. Souza, Clébson S. Tavares, Silverio O. Campos, Raul N.C. Guedes, Eliseu J.G. Pereira

**Affiliations:** 1 Departamento de Entomologia, Universidade Federal de Viçosa, Viçosa, MG, 36570-900, Brazil; 2 Instituto Nacional de Ciência e Tecnologia em Interações Planta-Praga, Universidade Federal de Viçosa, Viçosa, MG, 36570-900, Brazil

## Abstract

Transgenic crop “pyramids” producing two or more *Bacillus thuringiensis* (Bt) toxins active against the same pest are used to delay evolution of resistance in insect pest populations. Laboratory and greenhouse experiments were performed with fall armyworm, *Spodoptera frugiperda*, to characterize resistance to Bt maize producing Cry1A.105 and Cry2Ab and test some assumptions of the “pyramid” resistance management strategy. Selection of a field-derived strain of *S. frugiperda* already resistant to Cry1F maize with Cry1A.105 + Cry2Ab maize for ten generations produced resistance that allowed the larvae to colonize and complete the life cycle on these Bt maize plants. Greenhouse experiments revealed that the resistance was completely recessive (*Dx* = 0), incomplete, autosomal, and without maternal effects or cross-resistance to the Vip3Aa20 toxin produced in other Bt maize events. This profile of resistance supports some of the assumptions of the pyramid strategy for resistance management. However, laboratory experiments with purified Bt toxin and plant leaf tissue showed that resistance to Cry1A.105 + Cry2Ab2 maize further increased resistance to Cry1Fa, which indicates that populations of fall armyworm have high potential for developing resistance to some currently available pyramided maize used against this pest, especially where resistance to Cry1Fa was reported in the field.

Transgenic crops producing toxins from the soil bacterium *Bacillus thuringiensis* (Bt) are a major tool for controlling insect pests worldwide^1^. Benefits of Bt crops include effective management of target pests, decreased use of conventional insecticides, and reduced risks to non-target organisms, including humans^2^,^3^,^4^,^5^,^6^. However, rapid evolution of resistance to Bt crops in several pest species has compromised some of these benefits^7^,^8^.

To increase pest control efficacy and delay resistance evolution, a gene pyramiding strategy has been employed in transgenic plants to produce two or more Bt toxins of dissimilar mode of action effective against the same target pest species^7^. As a result, pyramided Bt crops have been adopted rapidly and are expected to become even more prevalent in the future^9^. As a strategy for resistance management, pyramided Bt crops are expected to be more effective when insect resistance to each Bt toxin is recessive and associated with reduced fitness of resistant individuals on both non-Bt and Bt plants (i.e., with fitness costs and incomplete resistance), when selection with any one of the pyramided toxins does not cause cross-resistance to the others, and when alleles conferring resistance to each toxin are rare^10^,^11^,^12^,^13^. However, little is known about that evolutionary response from target species to the selective pressure imposed by two or more toxins with independent mechanisms of action.

In this study we characterize the inheritance of resistance to pyramided maize producing the Cry1A.105 and Cry2Ab toxins in the fall armyworm, *Spodoptera frugiperda*, a major migratory pest of maize in Neotropical America^14^, and recently also in southern United States^15^. Previous studies with resistant strains of *S. frugiperda* to the event TC1507 (i.e., non-pyramided Bt maize producing the Cry1Fa toxin) showed that the genetic basis of resistance did not fit some assumptions of the high dose-refuge strategy, i.e., lack of fitness costs associated with resistance, high frequency of resistance alleles, and heterozygote survival greater than 5% on leaf tissues of Cry1F maize plants^16^,^17^. The result has been the rather quick appearance of Cry1F resistance in Brazilian populations of the fall armyworm^16^,^18^.

An additional concern regarding the two-toxin pyramiding strategy is that previous exposure and selection for pest resistance to single-toxin Bt crops containing one of the pyramided toxins may facilitate evolution of resistance to the two-toxin Bt event^7^,^10^,^11^,^13^,^19^. The concurrent cultivation of single-gene Bt maize plants producing one of the pyramided toxins or a toxin acting by same mode (i.e., sharing the same binding site in receptor proteins) and the two-gene Bt plants will potentially lead to the same outcome – fast evolution of pest resistance to the two-toxin pyramided event^7^,^11^,^13^. As Bt maize cultivation in Brazil exhibits both situations, the likelihood of quick evolution of resistance to two-toxin Bt pyramided events in the fall armyworm, the key maize pest species in the country, seems high although not yet assessed.

To our knowledge, this is the first study that assess whether the evolutionary response of a target species meets the conditions that favor the durability of pyramided Bt crops. We found that the resistance to pyramided maize was completely recessive (*Dx* = 0), incomplete, autosomal, and without maternal effects or cross-resistance to the Vip3Aa20 toxin produced in other Bt maize events. Although this profile is favorable for resistance management, selection for resistance to Cry1A.105 + Cry2Ab reduced the susceptibility to Cry1Fa, indicating cross-resistance between these toxins. This finding raises concern regarding the sustainable use and efficacy of pyramided maize hybrids in countries such as United States and Brazil, where the frequency of Cry1Fa resistance alleles in *S. frugiperda* is high,^16^,^20^,^21^ and many of the pyramided maize hybrids available produce Cry1A, Cry1F, and Cry2A^9^.

## Results

### Selection for resistance to Cry1A.105 + Cry2Ab maize

**Figure 1:**
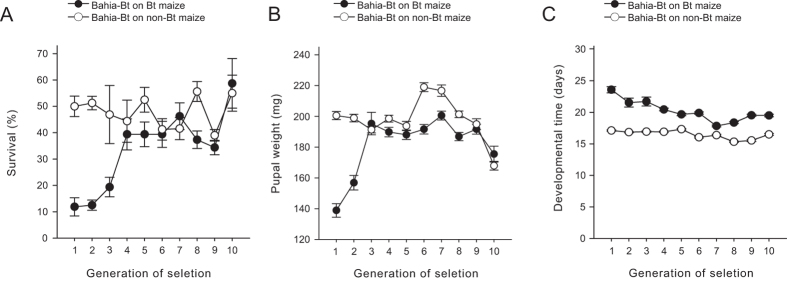
Response to selection for resistance to a dual-gene Bt maize event in the fall armyworm, *S. frugiperda*. Data are means ± standard errors. (**a**) Survival from neonate to pupa, (**b**) pupal weight, and (**c**) developmental time from neonate to adult for the selected *S. frugiperda* strain reared on maize leaves of the event MON89034 (–●–) or its non-transgenic isoline (–○–) during 10 generations of selection for resistance to event MON89034, which produces Cry1A.105 and Cry2Ab proteins from *B. thuringiensis*.

Larvae under exposure to the pyramided Bt maize responded to selection for resistance to Cry1A.105 and Cry2Ab proteins, as indicated by increased survival, pupal biomass and a decrease in development time from neonate to adult during the selection experiment (Fig. 1). Survival rates for individuals reared on transgenic maize increased until the fourth generation of selection, and then kept constant above 35%; in the later generations of selection, the selected strain was surviving at rates similar to those of control individuals reared in non-Bt maize (*P* > 0.05) (Fig. 1a). The response to selection pressure was also evident in the weight of the pupae exposed to the transgenic event, with significant gain in weight until the third generation. Afterwards in the F_9_ and F_10_ generations, pupal weight of the selected insects did not differ from the weight of individuals reared on non-Bt maize (*P* < 0.05) (Fig. 1b). The development time from neonate to pupa decreased from the first generation of selection (F_1_) to the last one (F_10_), although at the end of the selection experiment, individuals fed in the transgenic maize still required three days more than those fed on non-Bt maize to reach the adult stage (Fig. 1c).

### Fitness of selected insects on Bt transgenic plants

**Figure 2:**
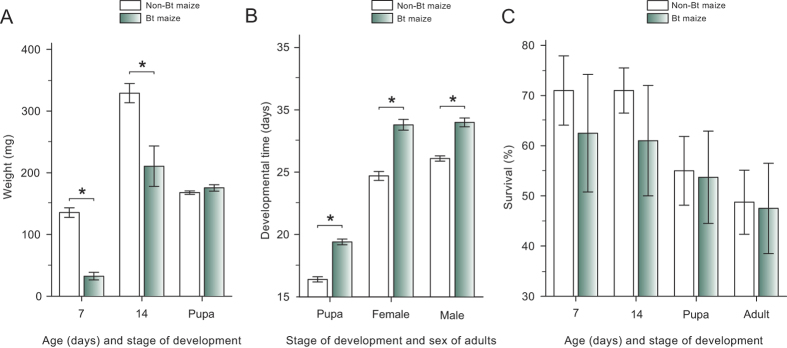
Life-history traits of Cry1A.105 +Cry2Ab-selected fall armyworm on leaves of Cry1A.105 + Cry2Ab Bt maize or its non-Bt isoline. Data are means ± standard errors measured after 10 generations of selection for resistance to event MON89034, which produces Cry1A.105 and Cry2Ab. (**a**) Larval weight at 7 and 14 days of post-embrionic development and in the pupa stage. (**b**) Developmental time from neonate to pupa and to the adult stage (male and female). (**c**) Survival in the larva stage at 7 and 14 days, and at the pupa and the adult stages. Bars with asterisk indicate significant differences (Fisher´s protected LSD, *P* < 0.05) between maize types.

Life-history traits of individuals of the selected strain reared on non-Bt or Bt maize producing Cry1A.105 and Cry2Ab are shown in Fig. 2. Larval weights at 7 and 14 days for individuals reared on non-Bt maize were significantly higher than those for individuals reared on the Bt maize (*P* < 0.05). However, the pupal weight was similar in both non-Bt and Cry1A.105 + Cry2Ab maize. Developmental time from neonate to pupa and to adult (both sexes) were significantly lower for individuals reared in non-Bt maize than for individuals reared on the Bt plants (*P* < 0.05); on average, individuals exposed to Cry1A.105 + Cry2Ab maize had a 3-day delay to reach the pupa and adult stages. No difference was observed between males and females reared on the same cultivar (*P* > 0.05) (Fig. 2b). Regarding survival rates, no differences between the two types of maize plants were obtained for larva, pupa or adult stages (Fig. 2c).

The number the female offspring produced per parental female (*R*_*o*_, i.e., the net reproductive rate) of the selected strain was significantly lower (*P* < 0.05) for individuals reared on Bt maize than for those reared on non-Bt maize (Table 1). Similar results were obtained in the intrinsic rate of population increase (r_m_) (Table 1), indicating that the potential population growth of the selected strain is lower on the transgenic maize than on non-transgenic one. Additionally, the mean generation time (*T*) and time for the population to double its size (*Dt*) were significantly higher for the cohort reared on the Bt maize (Table 1), confirming the trend of lower fitness of the resistant insects in the transgenic maize and thus indicating that the resistance to Cry1A.105 + Cry2Ab maize in this strain of *S. frugiperda* is incomplete.

### Susceptibility to Cry1Fa

As indicated by *P*-values higher than 0.05 (Table 2), the probit model fitted the data well allowing valid estimates of the parameters that describe the susceptibility of the strains to Cry1Fa. The highest concentration tested in the bioassays was 30,000 ng.cm^−2^, which did not cause any death of neonates of the selected strain (Table 2). Based on this concentration we estimated the lower limit of the resistance ratio, which was >12-fold greater than the concentration required to kill 50% (LC_50_) of neonates of Bahia-Cv strain (Table 2) and represents a conservative estimate of the resistance ratio. Similarly, the concentration required to inhibit 50% growth (EC_50_) of larvae of the selected strain was 500-fold greater than the EC_50_ value of the larvae of Bahia-Cv strain (Table 2). These results show that after ten generations of selection with maize producing Cry1A.105 + Cry2Ab toxins, the selected strain exhibited lower susceptibility to Cry1Fa than the Bahia-Cv strain maintained without exposure to Cry1A.105 + Cry2Ab maize during the selection experiment.

### Inheritance of resistance

**Figure 3:**
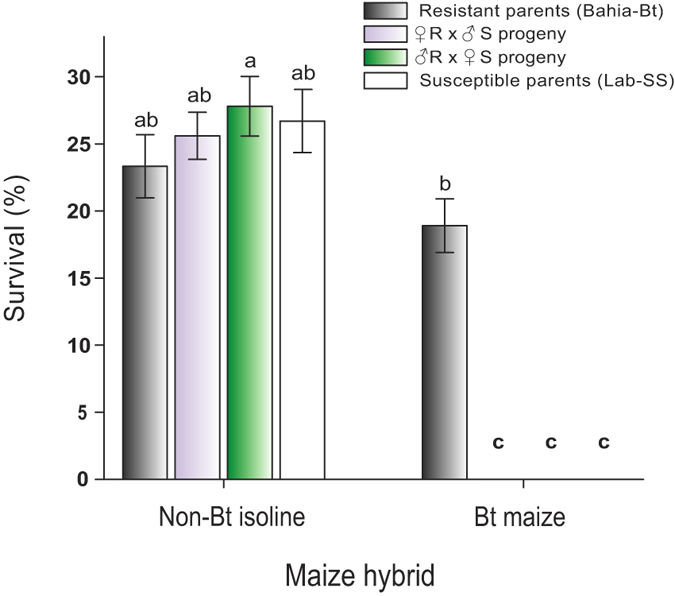
Functional dominance of resistance to a dual-gene Bt maize event in the fall armyworm, *S. frugiperda*. Data are means ± standard errors of survival rates to adulthood when feeding on vegetative stages of maize plants from event MON89034 (producing Cry1A.105 + Cry2Ab) or its non-Bt isoline. Survival was measured for a susceptible control strain (Lab-SS), the selected strain (Bahia-Bt) and progenies from reciprocal crosses between the two strains. Different letters above bars indicate significant differences among *S. frugiperda* genotypes (Fisher´s protected LSD, *P* < 0.05) and between maize hybrids.

Susceptible, F_1_, and resistant individuals responded differently to non-Bt and Cry1A.105 + Cry2Ab maize as indicated by the significant interaction in the analysis of variance (*F*_3,56_ = 21.64, *P* < 0.001). The survival of the parental strains and heterozygotes resulting from reciprocal crosses (R♀ × S♂ and R♂ × S♀) was significantly different on the transgenic Cry1A.105 + Cry2Ab maize (*F*_7,64_ = 57.7, *P* < 0.001). The susceptible strain (Lab-SS) and heterozygous offspring died within seven days, unlike individuals of the Bahia-Bt strain, which completed larval development and reached the adult stage (Fig. 3). These results show that the inheritance of resistance in the selected strain is completely recessive (*D*_*x*_ = 0), with heterozygotes exhibiting survival rates similar to those shown by the susceptible strain in isogenic non-Bt and Cry1A.105 + Cry2Ab maize cultivars (Fig. 3).

### Cross-resistance

**Figure 4:**
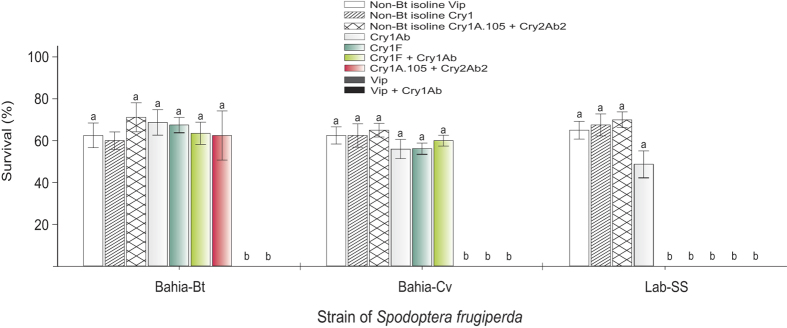
Resistance and cross-resistance to Bt maize events in the fall armyworm, *S. frugiperda*. Data are means ± standard errors of survival rates at seven days for three *S. frugiperda* strains on transgenic Bt maize events of first and second generation. In the legend, the maize hybrid, Bt event, or Bt protein (maize product) used were as follows: *Isoline Vip*, Syngenta Status; *Isoline Cry1*, Pioneer 30F53; *Isoline Cry1A.105 + Cry2Ab*, Monsanto DKB390; *Cry1Ab*, event MON810 (Pioneer 30F53Y); *Cry1F*, event TC1507 (Pioneer 30F53 H); *Cry1F* + *Cry1Ab,* events TC1507 + MON810 (Pioneer 30F53 HY); *Cry1A.105 + Cry2Ab*, event MON89034 (Monsanto DKB390PRO2); *Vip*, event MIR162 (Syngenta Status Viptera); *Vip + Cry1Ab*, events MIR162 + Bt11 (Syngenta Status TL Viptera). Different letters above bars indicate significant differences among maize hybrids (Tukey´s HSD, *P* < 0.05).

Survival rates for Bahia-Bt, Bahia-Cv and Lab-SS strains reared on transgenic maize hybrids are shown in Fig. 4. There was significant interaction between strain of *S. frugiperda* and maize hybrid (*F*_16,__182_ = 18.94, *P* < 0.001), indicating that *S. frugiperda* survival is dependent on the maize hybrid. The Lab-SS strain showed similar survival to that of the selected (i.e., Bahia-Bt) and control (i.e., Bahia-Cv) strains (*P* > 0.05) on the three non-Bt maize hybrids and that producing Cry1Ab. In contrast, 100% mortality was observed for the susceptible fall armyworm strain (i.e., Lab-SS) when feeding on the other transgenic maize hybrids, thus indicating the high potential of control efficacy of these transgenic hybrids against *S. frugiperda*. Nevertheless, survival of the non-selected control Bahia-Cv strain on Cry1A.105 + Cry2Ab maize was lower than that of the selected Bahia-Bt strain, and on other maize hybrids tested the survival rates between selected and non-selected control strains were similar. Importantly, survival of the Bahia-Cv strain on Cry1F maize was significantly higher than that of Lab-SS on this Bt maize, showing that field-evolved resistance to Cry1F had already occurred before selection of Bahia-Bt on Cry1A.105 + Cry2Ab maize. Finally, the Bahia-Bt strain showed 100% mortality in maize hybrids producing Vip3Aa20 (MIR162 and Bt11/MIR162), indicating that selection for resistance to Cry1A.105 + Cry2Ab maize did not decrease susceptibility to events producing Vip3Aa20.

## Discussion

The work described here demonstrates that *S. frugiperda* responds to selection for resistance to dual-gene Bt transgenic maize producing Cry1A.105 and Cry2Ab. This was evident by the consistent increases in larval survival and pupal biomass as well as decrease in developmental time over the generations of selection for resistance to Cry1A.105 + Cry2Ab maize. The significant level of resistance evolved by the selected strain was evidenced by its ability to complete larval development on Cry1A.105 + Cry2Ab maize plants in the greenhouse. Such response to selection for resistance to dual-gene transgenic maize is similar to the response obtained in two different strains of *S. frugiperda* selected for resistance to the single-gene maize producing Cry1Fa^16^. The similarity in response to selection in *S. frugiperda* between pyramided and non-pyramided events contrasts with the prediction that transgenic crops expressing two or more toxin genes would be more effective in delaying resistance evolution than single Bt-toxin crops^11^,^19^,^22^.

In our study, larval survival rates of the non-selected (i.e., Bahia-Cv) strain did not differ on leaves of non-Bt and Cry1F maize plants, and these Bt plants did not kill the Bahia-Cv larvae while killing all the larvae of a standard susceptible strain (see Fig. 4). More importantly, the LC_50_ value for larvae of the Bahia-Cv strain shown in Table 2 was greater than those of the LC_99_ established as diagnostic concentrations for Cry1F resistant individuals (i.e., 2000 ng.cm^−2^ or 1870 ng.cm^−2^)^16^,^18^. Because Cry1F and Cry1A.105 are similar toxins sharing binding sites in receptor proteins in the insect midgut^23^, cross-resistance between Cry1F and Cry1A.105 is expected in *S. frugiperda*. In fact, our results indicate cross-resistance between Cry1F and Cry1A.105 because selection with Cry1A.105 + Cry2Ab maize increased resistance to Cry1F in the selected strain relative to unselected strain (see Fig. 4). Therefore, the rapid response to selection with Cry1A.105 + Cry2Ab maize is partly explained by the fact that the selected and unselected strains were derived from individuals collected on Cry1F maize that were resistant to Cry1F maize before selection started, that is, Cry1A.105 + Cry2Ab maize was more like a single-toxin crop at the onset of selection. This raises concerns regarding the concurrent deployment of Cry1F and Cry1A.105 + Cry2Ab maize because of the cases of Cry1F resistance documented in Brazil and the United States^18^,^21^, and the fact that *S. frugiperda* moths are highly mobile and even migratory in at least some regions of the distribution range.

Even with evidence of shared binding sites for Cry1A.105 and Cry1Fa but not Cry2Ab in *S. frugiperda*^23^ as well as cross-resistance between Cry1A.105 and Cry1Fa^21^ but not between Cry2Ab and Cry1Fa^21^, it is still possible that correlated resistance to Cry2Ab and Cry1Fa may partly explain the rapid response to selection for resistance to Cry1A.105 + Cry2Ab maize here observed. Future studies should test whether the Cry2Ab toxin alone affects survival of larvae resistant to Cry1A.105+Cry2Ab maize. In addition, as some Cry1 and Cry2 Bt toxins have structural homology in domain II^9^, substantial levels of cross-resistance can occur between them^9^,^24^ and more work is needed to determine whether there is cross-resistance between Cry1Fa and Cry2Ab in *S. frugiperda*.

In terms of resistance management applied to transgenic Bt plants, the results of this investigation point out the risky deployment of maize producing Cry1A.105, Cry1Fa and Cry2Ab against *S. frugiperda*, especially where Cry1Fa resistance has already been reported in the field and the frequency of resistance alleles is high.^16^,^18^,^20^,^25^. This implication follows from the likely cross-resistance between Cry1A.105 and Cry1Fa such that pyramided maize containing Cry1A.105 + Cry2Ab or Cry1A.105 + Cry1Fa + Cry2Ab may only exert effective control against the fall armyworm with a single protein (i.e., Cry2Ab), making them as vulnerable to resistance as single-toxin Bt maize from the first generation. A similar scenario with Bt cotton was anticipated for cotton bollworm in Australia^26^.

Interestingly, when feeding on Cry1A.105 + Cry2Ab maize, larvae of the selected strain had lower body weight and a 3-day delay in developmental time, but these effects did not reduce pupal weight or adult survival, fitness components that were similar to those of insects reared in the non-Bt maize. However, these sublethal effects on larval weight and development time had negative consequences for adult fitness as evidenced in the net reproductive rate (*R*_*o*_) and intrinsic rate of population increase (*r*_*m*_), demographic parameters that describe the potential population growth of the selected strain (see Table 1). Because fitness estimates (i.e., *R*_*o,*_*r*_*m*_) were lower for resistant individuals reared in transgenic maize leaf tissue, the resistance in Bahia-Bt strain is characterized as incomplete^27^.

For pyramided Bt crops, incomplete resistance may have more complex effects on the rate of resistance evolution than anticipated^1^,^28^. While adapting to a toxin in the pyramid, individuals are exposed to the other toxin and may be killed and/or experience sublethal effects compromising their fitness, as shown here and elsewere^28^. Despite the substantial effect that incomplete resistance may have to delay resistance evolution^1^,^28^, delayed larval development resulted in three days of asynchronous emergence of the adults reared in transgenic maize leaves relative to those from non-Bt maize (Fig. 2b), which could lead to unsuccessful matting between resistant and susceptible individuals^29^. If this occurs under field conditions, resistant *S. frugiperda* moths may mate among themselves more often than with susceptible moths from refuges, thus making resistance management more challenging.

In the greenhouse, mortality of heterozygotes from reciprocal crosses between susceptible and resistant parents (R♀ × S♂ and R♂ × S♀) indicated that the inheritance of the resistance to Cry1A.105 + Cry2Ab maize is completely recessive (*Dx* = 0), autosomal, and without maternal effects. Assuming that two loci contribute to resistance (i.e., locus 1 confers resistance to Cry1A.105 and locus 2 to Cry2Ab), the recessive inheritance observed indicates recessive resistance to Cry1A.105, Cry2Ab, or both. Thus, further research is needed to assess dominance of resistance to each toxin. To our knowledge, this is the first characterization of the resistance of *S. frugiperda* to Bt maize producing two toxins, which in part has been consistent with previous investigations using fall armyworm strains with high levels of resistance to first generation Bt maize under field conditions^18^,^25^, and in the laboratory^16^.

This pattern of functionally recessive resistance showing 100% mortality of heterozygotes suggests that Cry1A.105 + Cry2Ab maize produces high concentration of Bt protein against *S. frugiperda*, providing support for this key assumption of the pyramiding strategy^9^,^11^,^13^. However, further experiments with Bt maize plants producing Cry1A.105 alone, Cry2Ab alone, and both toxins would be needed to identify if any of these toxins meet the high dose criterion. Additionally, in order to devise appropriate resistance management tactics, further research is needed to investigate if fitness costs are higher in insects exhibiting resistance to crops producing multiple toxins, which could also contribute to the rarity of resistance alleles. All this information is especially important given that transgenic cultivars with two or more Bt toxins targeting individual pests are likely to become increasingly prevalent^9^ and the conditions that favor durability of pyramided Bt crops have not been sufficiently assessed.

Comparison of survival rates before (i.e., Bahia-Cv strain) and after the selection experiment (i.e., Bahia-Bt strain) showed that larval survival of the Bahia-Bt strain in maize producing Cry1Ab, Cry1F, Cry1F + Cry1Ab, Vip3Aa20 + Cry1Ab and Vip3Aa20 toxins did not differ significantly, indicating that selection with Cry1A.105 + Cry2Ab maize did not increase resistance to these Bt events (see Fig. 4). Nonetheless, after 10, as shown in Fig. 1 generations of selection, LC_50_ and EC_50_ values to Cry1F exhibited significant increase (see Table 2). All of these results suggest that adaptation to Cry1A.105 + Cry2Ab during the selection experiment led to lower susceptibility to Cry1F, but not to Vip3Aa20-producing maize, indicating the existence of some level of cross-resistance between Cry1A.105 and Cry1Fa but not between Cry1A.105 or Cry2Ab and Vip3Aa20, thus corroborating what was reported elsewhere^21^,^30^. That the susceptibility to Vip3A maize was sustained in the resistant insects here selected is likely due to the independent mechanisms of action of Cry1, Cry2, and Vip proteins^23^,^31^. This is consistent with recent studies with Cry1F-selected *S. frugiperda*^16^ and Vip3Aa20-resistant *H. armigera*^30^, in which no cross-resistance between Vip3A and Cry1 and Cry2 was observed. However, low levels of cross-resistance between Vip3A and Cry1A were suggested^9^, and additional work is needed to determine if cross-resistance exists between these toxins using Cry1A.105+Cry2Ab-resistant insects because lack of shared binding sites is not sufficient to infer cross-resistance among Bt toxins^9^,^32^.

The high mortality caused by Vip3Aa20 maize to larvae of the Bahia-Bt strain and of other Cry1F-resistant fall armyworm strains indicates the feasibility of using Vip toxins against Cry-resistant insects. This may be one of the few alternatives to manage *S. frugiperda* populations using transgenic Bt cultivars. However, as shown in this study, *S. frugiperda* has high capacity to adapt to pyramided maize events, as the risk of resistance evolution increases when there is cross-resistance between toxins produced by pyramided maize and those already showing field-relevant resistance (i.e., Cry1F in Brazil and United States). Therefore, caution should be taken when devising and market-releasing pyramided transgenic events producing Vip3Aa or Cry2Ab with other Cry1 toxins, especially where field resistance to Bt toxins has already been reported.

To our knowledge, this is the first study that documents the evolutionary response in an economically important insect, the armyworm *S. frugiperda,* to the selective pressure imposed by two *B. thuringiensis* toxins produced by a transgenic maize event. We have shown that a field-derived strain collected in the state of Bahia (Brazil) responded to selection pressure for resistance to the Cry1A.105 + Cry2Ab maize event. The response to selection was similar to that obtained previously with the Cry1F single-gene maize event, most likely due to field-evolved resistance to Cry1F in the strain used for selection of resistance to Cry1A.105 + Cry2Ab2 maize and the presence of cross-resistance between Cry1F and Cry1A.105. Interestingly, the observed resistance inheritance (completely recessive and incomplete) and the rate of 100% of mortality obtained for heterozygotes in Cry1A.105 + Cry2Ab maize is consistent with some of the assumptions of the resistance management strategy currently in place. However, the likely cross-resistance between Cry1F and Cry1A.105 raises concerns about the deployment of maize events producing Vip3Aa20 or Cry2Ab with Cry1A and Cry1F proteins. The availability of our selected strain simultaneously showing resistance to Cry1A.105 and Cry2Ab provides opportunities for conducting further studies on cross-resistance to other Bt proteins and on the costs associated with resistance to pyramided Bt plants, which will help in choosing suitable insecticidal proteins for pyramiding resistance genes in Bt maize hybrids against the fall armyworm. Moreover, the selected strain will allow for genetic, biochemical and molecular characterization of the resistance, which may also assist in refining recommendations for managing resistance to Bt toxins in *S. frugiperda*.

## Methods

### Insect collection and rearing

Larvae of *S. frugiperda* were collected during 2013 from Cry1F maize fields in the county of Luís Eduardo Magalhães, State of Bahia, Brazil. The insects collected were placed in trays with artificial diet^33^, packed in styrofoam boxes and sent to the Federal University of Viçosa (UFV). The moths resulting from these individuals were placed in PVC cages 40 cm high ×30 cm in diameter with sulfite paper on the inner walls for oviposition; cotton soaked in a solution of 10% sugar and 5% ascorbic acid was provided as food. Eggs were collected every other day for four days and stored in plastic bags until hatching. Batches of neonates were transferred to artificial diet^33^ in 500-ml plastic cups until the 2^nd^ instar, and then individually placed in 16-well PVC trays (Advento do Brasil, Diadema, SP) until pupation. The insects were kept at controlled temperature of 27 ± 2 °C, relative humidity of 70 ± 15% and 14L: 10D photoperiod.

### Selection for simultaneous resistance to Cry1A.105 and Cry2Ab

The experiment was conducted from October 2013 to September 2014 using maize leaves with the event MON89034 (DKB390PRO2, Monsanto Brazil, São Paulo, SP), which produces proteins Cry1A.105 and Cry2Ab2 from *B. thuringiensis* (hereinafter called Cry1A.105 + Cry2Ab maize). As control, we used the maize hybrid DKB390, a non-Bt isoline. Maize was sown every two weeks in 14-L pots in the experimental field keeping four plants per pot after thinning. Plants were watered twice a day and fertilized on days 10 and 35 with 40 g NPK (08–28–16) per pot. Crop cultivation practices were used as recommended for maize in the region, except for using manual weed control and no pesticide application.

Before the selection for resistance to Cry1A.105 + Cry2Ab maize, the base population of the fall armyworm was divided into two sub-populations or strains: one control, called Bahia-Cv, maintained in the absence of exposure to any Bt toxin, and the other called Bahia-Bt, to be selected for resistance to Cry1A.105 + Cry2Ab maize. The selection was performed in two phases using maize leaves in the stages V4-V9 (i.e. three to nine true leaves). In the first phase, from the 1^st^ to the 3^th^ generation of selection, a large number of neonates of age <24 h hatching were transferred to 500-ml plastic cups containing leaf pieces of Cry1A.105 + Cry2Ab maize. After 72 h, the survivors were transferred individually to plastic trays with artificial diet, where they were maintained until pupation. In the second phase of the selection experiment (4^th^ to 10^th^ generation), batches of 10 neonates were transferred into each well of a 16-well PVC tray (Advento do Brasil, Diadema, SP) containing 340 mg of leaf sections of either Cry1A.105 + Cry2Ab maize or its non-Bt isoline. After seven days, the larvae were individualized to avoid death by cannibalism, and the food was changed daily until pupation^16^. In each generation of selection, two types of maize (Bt and non-Bt isoline) were compared in terms of survival from neonate to pupa, pupal weight and development time from neonate to pupa, using 32 replicates (tray wells) per type of maize.

### Fitness of selected insects on Cry1A.105 + Cry2Ab maize

In the laboratory, 160 larvae of the selected strain were reared to pupation on leaf pieces of Cry1A.105 + Cry2Ab maize and its non-transgenic isoline using the same procedures described in the selection experiment. As fitness components, we recorded larval weight and survival at seven and 14 days, as well as weight and survival of pupae and adults. Developmental time from neonate to pupa and to adult were also recorded. Other life-history traits compared were those related to the population growth potential, which were determined using the life table format as described elsewere^34^,^35^ using the SAS statistical package^37^. Life table statistics estimated were the net reproductive rate (female offspring production per parental female, *R*_*o*_), intrinsic rate of population increase (daily female offspring production per parental female, *r*_*m*_), generation time (*T*), and the doubling time (time span necessary for doubling the initial population, *Dt*)^37^,^38^.

### Susceptibility to Cry1Fa

We tested the hypothesis that selection for resistance to Cry1A.105 + Cry2Ab maize in *S. frugiperda* decreases its susceptibility to Cry1Fa as it competes with Cry1A.105 for binding sites in fall armyworm brush border membrane vesicles^23^, leading to cross-resistance. Concentration-response bioassays were performed with the Bahia-Bt (after the selection experiment) and the Bahia-Cv strain (maintained in the absence of selection pressure since its establishment as a laboratory colony).

We used diet surface bioassays as described by Marçon *et al.*^39^ with slight modifications^20^. To establish the concentration of toxin that causes 50% mortality (LC_50_) or 50% growth inhibition (EC_50_), we used seven graded concentrations of Cry1Fa purified protein plus a control. Dilutions were prepared in 0.1% Triton X-100 to obtain uniform spreading on the surface of the diet. After air drying at room temperature, a single neonate was transferred to each well, and the bioassay trays were kept in an incubator with scotophase of 24 h at 27 °C and 70% relative humidity. Mortality was assessed after seven days of exposure. Larvae that were unable to molt to the second instar or weighed less than 0.1 mg were considered dead^39^. The weights of surviving larvae were recorded to determine the percentage of growth inhibition relative to controls. For each strain, the bioassays were repeated twice on two different dates, using 16 neonates per concentration. The Cry1Fa toxin used in the bioassays was obtained from the laboratory of Dr. Marianne P. Carey (Case Western Reserve University, OH). The protein was activated with trypsin (HPLC purified), supplied in the lyophilized form, and stored at −80 °C. The toxicity of the Cry1F stock used in the bioassays with *S. frugiperda* is similar to that obtained in other laboratories^20^,^25^.

### Determining the inheritance of resistance

The level of dominance was determined by comparing larval survival rates of Cry1A.105 + Cry2Ab-selected, control Lab-SS strains, and F_1_ heterozygous hybrids. The Lab-SS strain is a susceptible laboratory colony maintained without insecticide exposure for over 15 years at Embrapa Maize & Sorghum (Sete Lagoas County, State of Minas Gerais, Brazil). The heterozygous hybrid larvae were produced from reciprocal crosses between the Cry1A.105 + Cry2Ab selected and the control Lab-SS strains. To generate the F_1_ hybrids (R♀ × S♂ and R♂ × S♀), pupae of susceptible and selected strains were separated by sex based on morphological differences of the last abdominal segments. Fifty pupae of the opposite sex of each strain were placed in two mating cages made of PVC cylinders (40 cm high ×30 cm Ø). The survival of heterozygotes (R♀ × S♂ and R♂ × S♀) and parental strains in Cry1A.105 + Cry2Ab and non-Bt maize was evaluated in a greenhouse (28 ± 5 °C and 70% r.h.) using a completely randomized design. The maize hybrids were grown in 10-L pots (one plant/pot) and randomly placed in cages (1.5 × 3 ×2 m) framed by ½-inch-PVC pipes covered with voile fabric to reduce the risk of larval dispersal between treatments. Nine plants were placed in each cage and spaced 0.25 m within rows and 0.70-m between rows. Cultivation practices were the same described for the selection experiment. At the V4 stage, each plant was infested with 10 neonates, totaling 90 larvae per treatment and 180 per *S. frugiperda* genotype (Bahia-Bt, Lab-SS, R♀×S♂, and R♂×S♀). Fourteen days after infestation the plants were dissected, and the surviving larvae were counted and reared in the laboratory on leaves of the corresponding maize hybrid (Bt or non-Bt) until pupation and adult emergence.

The effective or functional dominance of resistance (*D*_*x*_) was calculated using the formula *D*_*x*_ = (X_RS_ – X_SS_)/(X_RR_ – X_SS_), where X_RR_, X_RS_ and X_SS_ are quantitative values of survival for homozygote resistant, heterozygote and homozygote susceptible strains, respectively^40^. *D*_*x*_ values can range from 0 (completely recessive resistance) to 1 (completely dominant resistance). When *D*_*x*_ is 0.5, the resistance is referred to as codominant or additive^40^. Sex-linked resistance was determined by comparing survival between the heterozygous strains in transgenic maize.

### Determining cross-resistance

We determined larval survival for Bahia-Bt, Bahia-Cv, and Lab-SS strains on six Bt maize hybrids. Three of them were single-protein Bt events: TC1507 (30F53H; Dupont Pioneer, Santa do Cruz do Sul, RS, Brazil), MON810 (30F53Y; Dupont Pioneer, Santa do Cruz do Sul, RS, Brazil), and MIR162 (Agrisure Viptera; Syngenta, São Paulo, SP, Brazil), which respectively produce Cry1Fa, Cry1Ab, and Vip3Aa20. The other three were pyramided Bt maize hybrids with the following events: MON89034 (DKB390PRO2, Monsanto do Brasil, São Paulo, SP), Bt11/MIR162 (Status Viptera, Syngenta, São Paulo, SP, Brazil), and TC1507/MON810 (30F53YH; Dupont Pioneer, Santa do Cruz do Sul, RS, Brazil), producing respectively the Bt proteins Cry1A.105 + Cry2Ab, Cry1Ab + Vip3Aa20, and Cry1Fa + Cry1Ab. Such Bt products represented those available commercially in Brazil to manage *S. frugiperda* (Comissão Técnica Nacional de Biossegurança, CTNBio, 2015). We used non-Bt isoline maize hybrids as controls: 30F53 (Dupont Pioneer, Santa do Cruz do Sul, RS, Brazil) for Cry1Fa-, Cry1Ab-, and Cry1Fa+Cry1Ab-maize products; Status (Syngenta, São Paulo, SP, Brazil) for Vip3Aa20 + Cry1Ab maize, and DKB390 (Monsanto do Brasil, São Paulo, SP) for Cry1A.105 + Cry2Ab Bt maize.

Plants were grown as described in the section on selection for resistance. At the V4-V5 stage, maize whorl leaves were excised, brought to the laboratory, and 340 mg of leaf sections were placed in each well (5.6 × 3.6 × 3 cm) of 16-well PVC trays (Advento do Brasil, Diadema, SP). Ten neonates (<24 h hatching) were transferred to each well using a fine hair brush. Larval survival was recorded daily, and leaf sections used as food were daily replaced until 7 d. Thereafter, leaf sections were replaced every 3 d. The experiment was performed under laboratory conditions (27 ± 2 °C, 70 ± 15% r.h., and 14L: 10D photoperiod) using a completely randomized design. Twenty seven treatments were combined in a factorial scheme: nine maize hybrids and three *S. frugiperda* strains. Each treatment had 16 replications for a total of 160 individuals evaluated in each maize hybrid.

### Statistical analyses

For each generation of selection, insects feeding on the two versions of maize (Bt and non-Bt isoline) were compared for survival rates from neonate to pupa, pupal weight, and developmental time from neonate to pupa. Similar data were gathered in the experiment of fitness of selected insects (i.e., incomplete resistance) and were subjected to analysis of variance and to (protected) Fisher’s least significant difference post-hoc test (LSD or t-test, *P* < 0.05) (SAS PROC GLM)^36^ when appropriate. For data on the population growth potential, life-table statistics were calculated as reported elsewere^37^ using the jackknife technique to estimate variance.

Survival data of hybrid progenies (R♀ × S♂ and R♂ × S♀) and parental strains in both versions of maize (Bt and non-Bt isoline) were subjected to two-way analysis of variance (four strains of *S. frugiperda* × two maize hybrids) followed by (protected) Fisher’s least significant difference post-hoc test (LSD or t-test, *P* < 0.05) (SAS PROC GLM)^36^. Before any analysis of variance run in this study, the assumptions of normality and homogeneity of variance were checked (SAS PROC MIXED followed by PROC UNIVARIATE, and PROC GPLOT)^36^, and no transformation was needed.

Data from the bioassays with purified Cry1Fa were subjected to probit analysis^41^ using PoloPlus^42^. Resistance ratios with 95% confidence limits were calculated based on mortality and growth inhibition data for the Bahia-Cv strain using PoloPlus^42^,^43^. Data obtained in the cross-resistance experiment were subjected to analysis of variance to compare insect survival among armyworm strains within each Bt maize hybrid, and subsequently to Tukey’s Honestly Significant Difference procedure for mean separation (*P* < 0.05) using the PROC GLM procedure of SAS^36^.

## Additional Information

**How to cite this article**: Santos-Amaya, O. F. *et al.* Resistance to dual-gene Bt maize in *Spodoptera frugiperda*: selection, inheritance, and cross-resistance to other transgenic events. *Sci. Rep.* **5**, 18243; doi: 10.1038/srep18243 (2015).

## Figures and Tables

**Table 1:** Incomplete resistance (i.e., fitness) of the selected fall armyworm strain on Cry1A.105 + Cry2Ab Bt maize.

Maize hybrid	Population growth statistic			
	*R*_*o*_	*r*_*m*_	*T*	*Dt*
Non-Bt	68.85 (39.2–98.4) a	0.167 (0.146–0.187) a	25.3 (24.3–26.4) a	4.12 (3.60–4.64) a
Cry1A.105 + Cry2Ab	27.63 (14.4–38.7) b	0.112 (0.095–0.129) b	29.8 (29.0–30.5) b	6.14 (5.18–7.10) b

Data are means and 95% confidence intervals for population growth statistics on two maize hybrids (*n* = 14–17 families).

*R*_*0*_, net reproductive rate (females per female per generation); *r*_*m*_, intrinsic rate of population increase (per day); *T*, mean generation time (days); *Dt*, doubling time, i.e., time for the population to double its size (days) (see Materials and Methods)^37^. Means (±95% confidence intervals) within columns followed by the same letter do not differ significantly (*P* > 0.05) through pairwise comparisons using two-tailed *t*-tests af*t*er the jackknife method to estimate variances associated with life-table statitics^37^.

**Table 2:** Susceptibility of the selected fall armyworm strain to the Cry1Fa protein from *B. thuringiensis.*

Response variable	Insect strain	Slope ± SE	LC_50_ or EC_50_ (95% CL)^a^	Resistance ratio (95% CL)^b^	*χ*^*2*^	*P*
Mortality	Bahia-Bt	nc^c^	>30000	>12	nc	nc
	Bahia-Cv	0.86 ± 0.08	2361 (1600–3792)	1	4.9	0.42
Growth inhibition	Bahia-Bt	2.84 ± 0.24	22407 (17200–32346)	549.3 (324.0–931.3)	0.5	0.99
	Bahia-Cv	0.93 ± 0.09	40.8 (24.9–60.4)	1	1.3	0.93

The protein was applied superficially onto the artificial diet and mortality and growth inhibition were recorded after seven days of exposure.

The number of insects tested in the bioassays was 251 and 506 for the Bahia-Bt and Bahia-Cv, respectively.

^a^Concentration killing 50% (LC_50_) or causing 50% growth inhibition (EC_50_), with 95% confidence intervals in parentheses^42^; units are in nanograms toxin per cm^2^ diet.

^b^Resistance ratio, the LC_50_ or EC_50_ value for the Bahia-Bt strain divided by the LC_50_ or EC_50_ values for the Bahia-Cv strain, with 95% confidence intervals in parentheses^42^.

^c^nc, not calculated, because of insufficient response even in the highest concentration tested (30,000 ng/cm^2^).
